# The Medical Institutional Repositories in Libraries (MIRL) Symposium: a blueprint designed in response to a community of practice need

**DOI:** 10.5195/jmla.2023.1503

**Published:** 2023-07-10

**Authors:** Brenda Fay, Lisa M. Buda, Anthony J. Dellureficio, Sara Hoover, Ramune K. Kubilius, Steven J. Moore, Lisa A. Palmer

**Affiliations:** 1 brenda.fay@aah.org, Library Manager, Midwest Region Library, Advocate Health, Milwaukee, WI, USA.; 2 lisa.buda@rochesterregional.org, Librarian, Libraries at Rochester Regional Health, Rochester Regional Health, Rochester, NY, USA.; 3 dellurea@mskcc.org, Associate Librarian, Data Management Services, Medical Library, Memorial Sloan Kettering Cancer Center, New York, NY, USA.; 4 shoover@gwu.edu, Metadata and Scholarly Publishing Librarian, Himmelfarb Health Sciences Library, The George Washington University, Washington, D.C., USA.; 5 r-kubilius@northwestern.edu, Collection Development / Special Projects Librarian, Galter Health Sciences Library & Learning Center, Northwestern University Feinberg School of Medicine, Chicago, IL, USA.; 6 smoore31@hfhs.org, Librarian, Sladen Library, Henry Ford Health, Detroit, MI, USA.; 7 lisa.palmer@umassmed.edu, Institutional Repository Librarian, Lamar Soutter Library, UMass Chan Medical School, Worcester, MA, USA.

**Keywords:** Institutional Repository, Scholarly Communication, Health Sciences Libraries, Medical Institutional Repositories in Libraries Symposium, Collaboration, Community of Practice

## Abstract

**Background:**

Health sciences libraries in medical schools, academic health centers, health care networks, and hospitals have established institutional repositories (IRs) to showcase their research achievements, increase visibility, expand the reach of institutional scholarship, and disseminate unique content. Newer roles for IRs include publishing open access journals, tracking researcher productivity, and serving as repositories for data sharing. Many repository managers oversee their IR with limited assistance from others at their institution. Therefore, IR practitioners find it valuable to network and learn from colleagues at other institutions.

**Case Presentation:**

This case report describes the genesis and implementation of a new initiative specifically designed for a health sciences audience: the Medical Institutional Repositories in Libraries (MIRL) Symposium. Six medical librarians from hospitals and academic institutions in the U.S. organized the inaugural symposium held virtually in November 2021. The goal was to fill a perceived gap in conference programming for IR practitioners in health settings. Themes of the 2021 and subsequent 2022 symposium included IR management, increasing readership and engagement, and platform migration. Post-symposium surveys were completed by 73/238 attendees (31%) in 2021 and by 62/180 (34%) in 2022. Feedback was overwhelmingly positive.

**Discussion:**

Participant responses in post-symposium surveys rated MIRL highly. The MIRL planning group intends to continue the symposium and hopes MIRL will steadily evolve, build community among IR practitioners in the health sciences, and expand the conversation around best practices for digital archiving of institutional content. The implementation design of MIRL serves as a blueprint for collaboratively bringing together a professional community of practice.

## BACKGROUND

Institutional repositories (IRs) focus on collecting, preserving, and disseminating the digital scholarship produced by an institution through its research and teaching. IRs also store other materials deemed worthy of preserving digitally or sharing, such as historical documents, marketing or promotional products, systematic review search methodologies, and supplementary data. Health sciences librarians around the world have identified IR adoption and maintenance as an important trend [1]. In the U.S. and Canada, IRs are well-established in medical schools and academic health centers [2] and have been adopted in health care networks and hospitals [3–4].

Institutions establish IRs to showcase institutional achievements in research, increase visibility, and expand the reach of institutional scholarship. IR content is generally open access for discovery and download around the world. A systematic review published in 2020 suggested that IRs have a positive impact on citation count and exposure [5], particularly for unique content not published in traditional venues, such as electronic theses and dissertations (ETDs), conference and meeting abstracts, preprints, and datasets. Digital repositories, including IRs, are now considered sources of literature for systematic reviews due to this unique content [6–7].

The role of IRs in health sciences libraries continues to evolve. Medical schools publish peer-reviewed open access journals and make open educational resources available through their IRs [2,8]. Library publishing extends to hospitals and health care networks, which utilize their IRs to publish peer-reviewed journals, as demonstrated by Advances in Clinical Medical Research & Healthcare Delivery from Rochester Regional Health and Journal of Maine Medical Center [9–10]. Repositories also serve as tools to track researcher productivity [3,11]. Recent developments to promote scientific data sharing have led to increased awareness of IRs. In January 2023, the National Institutes of Health implemented a data management and sharing policy for research data, with IRs and other generalist repositories identified as “potentially suitable data sharing options” [12].

Providing IR services requires a substantial investment in technology and staffing. IR managers often face numerous challenges, such as recruiting and harvesting content, determining policies, establishing workflows, copyright, and assessing the sustainability of repository operations. Many IR programs are staffed with one person or fewer [2,13]. Furthermore, the rate at which science journals publish increases more than 5% each year [14]. Repository managers, therefore, find it valuable to network and learn from colleagues at other institutions.

A number of conferences, user groups, and regional meetings have emerged that explicitly provide support for IR practitioners, including Open Repositories; Southern Miss Institutional Repository Conference (SMIRC); Digital Initiatives Symposium; Northeast Institutional Repository Day (NIRD); and Digital Commons North American Conference [15–19]. Castro et al. have documented how the NIRD conference was inspired and created to support the repository manager community in the Northeastern U.S. [20].

## CASE PRESENTATION

The purpose of this case report is to describe the genesis and implementation of a new health sciences initiative: the Medical Institutional Repositories in Libraries (MIRL) Symposium. We highlight the planning and outcomes of the inaugural 2021 symposium with additional insights from the subsequent 2022 event. We also discuss how we measured the success of the conference to inform future events and activities.

### Genesis and Implementation of a Born-Digital Symposium

Perceiving a dearth in programming specifically for medical institutional repositories, a hospital librarian approached a medical school librarian in early 2021 about launching an event for the health sciences IR community. They presented this idea to an invited group of librarians who agreed to discuss, plan, and execute a virtual meeting in late 2021 to address the perceived need. The final planning committee consisted of three U.S. hospital-based librarians and three U.S. academic health sciences librarians from five separate institutions in the East and Midwest.

Based on the committee members' experience, all of whom work in the institutional repository landscape in some capacity, no IR conferences were providing a specialized medical focus or track. Other repository conferences offer a multi-disciplinary, technical, or international suite of presentations. Although these conferences are useful to those involved in institutional repository work, case studies and best practices of IRs in medical institutions often become lost within these broader conference programs. We concluded that it was necessary to address that gap and to do so without significant financial overhead.

The goal was to organize a platform-neutral virtual symposium about IRs administered by libraries in academic medical centers, hospitals, and other healthcare settings. Our objectives included knowledge sharing, raising awareness of health sciences IRs, showcasing successful medical IR projects and initiatives, and forging connections between academic and hospital-based library practitioners.

Over a series of regularly scheduled virtual meetings in early-mid 2021, we chose a name–the Medical Institutional Repositories in Libraries (MIRL) Symposium–and developed a plan for a one-day conference that would feature a relevant keynote and a series of presentations. Given the global COVID-19 pandemic, we envisioned MIRL as a born-digital symposium. We intended for MIRL to be a free-of-charge event for participants, as many institutions were struggling with reduced staffing and professional development budgets. At the same time, we chose not to seek vendor sponsors, so that control over the program and service-provider influence remained entirely community-driven and at the discretion of the planning committee.

We minimized hosting costs by leveraging institutional licenses for virtual services that had emerged prior to and amid the pandemic. For example, a planning committee member hosted the conference on their institution's virtual conferencing platform. Another planning committee member offered to permanently host the symposium website in their IR. We designed a simple logo using a free trial for Canva.

We conducted planning activities through virtual meetings, cloud-based applications (Google Docs), and by email. As outlined in Table 1, tasks fell into several categories: administrative, marketing, technology solutions, keynote speaker, proposal workflow, communication, schedule, symposium day, post-conference, and evaluation.

**Table 1.** MIRL Symposium Planning Elements and Committee Activities

Planning Element	MIRL Planning Committee Activities
Administrative	Formed a committee with members of varied affiliations Chose a name, format, and duration for event Created a timeline and a shared task list hosted in Google Drive
Marketing	Identified discussion lists and other listservs for targeted emails Distributed save the dates, call for proposals, invitations to register Asked registrants and presenters to share symposium information with colleagues and networks Created symposium logo, images for website, and presentation slides using Canva
Technology Solutions	Chose technology resources: symposium website platform, video conference platform, proposal submission form, and post-conference survey form Enlisted institutional virtual platform technical support staff before and during the symposium Created plan for post-production and distribution of video recordings
Keynote speaker	Identified possible keynote speakers Voted on, selected, and invited keynote speaker
Proposal Workflow	Determined preliminary proposal formats Created proposal form using Google Forms Established criteria for proposal review process Scaled down length of all presentations due to high number of quality proposals
Communication	Convened monthly planning meetings (Microsoft Teams) Chose a single contact person to coordinate with keynote speaker Invited presenters to Zoom practice sessions prior to event
Schedule	In 2021 organized presentations and lightning talks by topic: Case reports; IR as a publishing platform; IR management and assessment; Data migration; Outreach and marketing; Pandemic-era initiatives; Showcases (vendors, providers, platforms); Supporting education In 2022 arranged sessions by content: Presentations central to IR audience first; Use cases and data-driven talks second; Novel presentations third
Symposium Day	Committee members volunteered as session moderators Created moderator templates for introductions, transitions, etc.
Post-Conference	Edited presentation recording into individual clips of presentations Enabled subtitles to be included in video files Archived video files and presentation slides on symposium website Created YouTube channel, @MIRLSymposium Shared link with presenters in postsymposium communication
Evaluation	Shared evaluation survey with registrants post-event Compiled results and discussed at committee meeting Incorporated responses into future planning

For the inaugural symposium, we decided our event would last only a few hours due to our uncertainty about the number of expected proposals. The schedule included a proposed keynote session that could address a timely and relevant topic. Inviting and confirming a keynote speaker early in the planning process allowed us to incorporate that information into the call for proposals and promotional announcements.

The call for proposals suggested various desired themes, such as collaboration, marketing, or operating an IR during a global pandemic. We solicited presentations and lightning talks with the hope that these two formats would provide ample opportunities to share information and generate more informal conversation. To provide repository managers with information about potentially valuable new tools and features, we reserved a small segment of the program for updates from service providers who work in the IR space.

Other action items included: deciding whether to record the sessions, setting up the registration, and reviewing the proposals. Establishing criteria for the submission review process was among the most important elements of the symposium planning process. We received 32 submissions in 2021 and 18 in 2022, and focused on topic, format, length, and relevance as the main evaluation criteria. We recommended changes in format for several presentations, but few were deemed off-topic or otherwise unsuitable for inclusion.

Moderators maintained contact with accepted speakers, provided follow-up instructions on technology issues, loaded presentation slides, and offered practice sessions. We also offered speakers a choice of pre-recording their presentation, rather than presenting live, though we expected them to join the symposium to field questions.

The final 2021 schedule consisted of 31 presentations in 5 timeslots: 1 keynote, 8 presentations of 12 minutes, 18 briefer talks of 5 minutes, and 4 vendor update presentations. The 2022 schedule was similar and included 1 keynote, 5 service provider update presentations, 10 lightning talks, and 3 presentations. The 2021 symposium panels were based on presentation type while the 2022 symposium was arranged by themes: starting, managing, and migrating an IR. For both symposia, we left time for Q&A at the end of each group of presentations.

### Measuring Success

To gain insight on how well we met our symposium objectives, we gathered event metrics such as the number of registrations and the number of submitted proposals, conversations from the lively virtual chat, and responses from a post-conference evaluation survey.

A total of 324 individuals registered for the inaugural event, including presenters and planning committee members. This exceeded the initial goal of 150 registrations. On the day of the symposium, 238 unique users participated in the event (75% of registrants). The second MIRL had 358 registrants including presenters and planning committee members, with 180 unique users participating on event day (50%).

The inaugural MIRL received a total of 33 proposal submissions, of which 31 were accepted. In 2022, we accepted all 18 proposal submissions. Each year, members of the planning committee also presented. During each symposium, communication through virtual chat increased after the keynote and remained steady throughout the day. Attendees and presenters communicated with each other, answering questions directly through chat. This was especially useful given the limited Q&A time.

We conducted a post-conference evaluation survey following the 2021 and 2022 events to learn more about registrants, publicity efforts, virtual platform use, and interest in symposium content. The survey was modified slightly from 2021 to 2022. The 2021 survey was completed by 73/238 attendees (31%). The 2022 survey was completed by 62/180 attendees (34%). Survey questions are in Supplemental File 1.

Participants indicated that MIRL met their expectations, with over 98% of respondents in both 2021 and 2022 indicating they were moderately or extremely satisfied with the program. Participants also suggested that the proposal formats were well designed, with the majority of respondents (56% in 2021 and 64% in 2022) indicating that presentation times were neither too long nor too short. The majority of respondents both years also found that the platforms used (Zoom in 2021 and WebEx in 2022) were adequate for the purpose. The majority of respondents after both symposiums (67% in 2021 and 72% in 2022) also indicated that MIRL should continue to be held annually.

The post-symposium survey also provided an open-response question inviting general comments from participants. Survey comments as a whole were overwhelmingly positive.

“Great event. I look forward to attending again in the future. As it was mentioned it is nice to know we aren't alone in the issues we face and to see how similar organizations dealt with the same issue.” (2021)

“The content was great. I heard from institutions solving similar problems in different ways. It was a great way to hear about what other institutions are doing.” (2022)

Feedback from open-ended survey questions fell into three categories: more breaks during the day, a wider variety of topics or IR platform representation, and fewer technical presentations.

“Very hard to sit for almost a solid 5 hours! Needed more breaks. Either all day with more break time allotted or 2 shorter days might be better.” (2021)

“It would have been interesting to hear about different (especially open access) IR platforms, such as Omeka, and perhaps some speakers from other countries!” (2021)

“The vendors were very technical. I felt like I needed a software engineering degree.” (2022)

Complete, de-identified Survey results associated with this article are in the Medical Institutional Repositories In Libraries (MIRL) Symposium website at https://hsrc.himmelfarb.gwu.edu/mirl/2021/program/39/ and https://hsrc.himmelfarb.gwu.edu/mirl/2022/program/21/.

Individual presentation videos, slides, and de-identified post-symposium survey results from the 2021 and 2022 symposia are archived in a single IR and can be viewed on the MIRL website [21]. The embedded videos from the 2021 symposium were streamed and captioned using Wistia [22]. The video recordings from the 2022 symposium are available on the MIRL YouTube channel [23]. As of May 2023, presentation slides from the two virtual events have now been downloaded over 600 times and have received nearly 2,000 metadata page hits by users at nearly 100 institutions in over 45 countries. Digital archiving allows new users to find symposium content and supports the planning committee's goal of building this community of practice.

## DISCUSSION

After the 2021 symposium, the planning committee reviewed the survey results and shared with each other their impressions of the successes and challenges of the inaugural event. We discussed scheduling, program flow, but most importantly, posed the question of whether MIRL should be organized again in the future, and if so, how often. Review of attendee feedback confirmed participant interest in an annual event and identified new volunteers for the planning committee. For 2022, two volunteers joined the planning group, while most of the original members stayed on, providing continuity. We proceeded to design a symposium that would build on the lessons learned from the 2021 inaugural event. MIRL 2022 also proved to be well received by the IR community.

Several themes emerged from the symposia presentations. The accepted proposals in 2021 fit into six categories: case reports (9), IR management and assessment (6), IR as a publishing platform (4), supporting education (4), outreach and marketing (3), and pandemic-era initiatives (3).

Proposals in 2022 exhibited a pattern the planning committee called the “life cycle of an IR”: the initial stage of evaluating and purchasing an IR platform (1), setting up and managing the IR (7), and migrating IR data to a new platform (5). Our strategy of first advertising an open invitation for proposals then curating the program has proven successful.

Notable changes from 2021 to 2022 include an increase from zero to five proposals on data migration and no proposals referencing the COVID-19 pandemic. In both years, many presenters chose to focus on IR management as their topic. Given the amount of time and energy required to operate and manage a repository, the majority of presentations were predictably concerned with practical considerations such as increasing readership and engagement, workflow tips, and reporting success and statistics to an IR's community. Based on survey responses, the community confirmed their appreciation for practical, “hands-on”, or “ground level” presentations that can be applied immediately by IR managers.

### Lessons Learned

The MIRL planning committee learned several valuable lessons while organizing the inaugural symposium.

Perhaps the most important was the value of having institutional technical support for the virtual meeting platform. The six professional librarians on the planning committee brought a variety of technical expertise and created numerous digital assets for the symposium. Nevertheless, we recommend exploring institutional IT resources and utilizing a dedicated specialist before, during, and after the event to resolve any technical issues without interfering with the committee members' other responsibilities. In 2022, a different committee member from the previous year managed the virtual platform, which necessitated changing the platform from Zoom to Webex. MIRL 2022 also utilized a dedicated technical support specialist, but the differences in software meant that the committee had to make adjustments from the previous year, such as reformatting the instructional email that was sent to presenters and reviewing some decisions about how to present the recordings.

Another practical takeaway from the symposium planning process was the importance of building in rest breaks. Even in a virtual environment, both participants and conference planners benefit from both short breaks and a limited number of sessions per panel. For MIRL 2022, the committee remained cognizant of this need and did their best to ensure more time was allotted for breaks and discussion. We also reminded attendees that the sessions would be recorded and posted on the MIRL website for later viewing if they needed to step away [21].

Each year the planning committee has used the survey responses to ensure MIRL continues to evolve and meet participants' expectations. Future changes may include adjustments in the format, length of presentations, vendor, and keynote offerings.

## FUTURE PLANS AND CONCLUSIONS

Based on two rounds of positive survey responses from participants, the MIRL planning committee decided to continue organizing the virtual symposium annually, evaluating and making revisions as necessary to stay relevant. As MIRL has met its goals of raising awareness, showcasing best practices, and forging connections among its participants, we felt it necessary to continue providing this opportunity for knowledge sharing to our community. The committee hopes that this event will continue to evolve, build connections between IR practitioners who work in the health sciences, and expand the conversation around best practices related to the digital archiving of institutional content. Recent repository developments, funder mandates, and an increasing global view may continue to expand the MIRL community in the future.

Developing community spaces through symposia, collaborations, communications networks, and other methods is never easy, more so when both the community and the need are perceived, rather than explicit. It requires dedication, planning, and adaptability on the part of a core group with common interests. As we have demonstrated, although this from-the-ground-up approach does require commitment, it does not necessarily require large funds, a robust organizational structure, or even a pre-existing community. The MIRL planning committee hopes that our experiences, methods, and logistics of converting a concept into a valued professional space with room for growth will provide a blueprint for others with similar goals.

## Data Availability

Data associated with this article are in the Medical Institutional Repositories in Libraries (MIRL) Symposium website at https://hsrc.himmelfarb.gwu.edu/mirl/2021/program/39/ and https://hsrc.himmelfarb.gwu.edu/mirl/2022/program/21/.
