# Supplementary material for: The Medical Institutional Repositories in Libraries (MIRL) Symposium: a blueprint designed in response to a community of practice need
Source: J Med Libr Assoc. 2023 Jul 10;111(3):710–6. doi: 10.5195/jmla.2023.1503 (PMC10361555; doi:10.5195/jmla.2023.1503)
Supplement: Supplementary file 1 — Appendix A [file jmla-111-3-710-s01.pdf]

Appendix A: MIRL 21/22
Supplemental File Survey Instrument

Q1 - Please indicate the type of organization with which you are affiliated. Check all that apply.

2021	2022
Academic archives	Academic archives
Academic health sciences library	
Academic archives	Academic archives
Academic library	Academic library
Corporate archives	
Corporate library	
Government	Government
Health care/Hospital library	Health care/Hospital library
Independent contractor	Independent contractor
Non-profit organization	Non-profit organization
Public library	
Student	Student
Student	Student
Vendor/Service provider	Vendor/Service provider
Other	Other

Q2 - What was your main motivation in attending MIRL Symposium 2021? Check all that apply.

2021	2022
Event topic	Event topic
Personal growth and development	Personal growth and development
Professional development	Professional development

Support the event and community	Support the event and community
Other	I attended for another reason

Q3 - How did you learn about MIRL Symposium 2021? Check all that apply.

2021	2022
AAHSL	
ACRL Health Sciences Interest Group	
	BLC Announce
CAMA-Chicago Area Medical Archivists	
Canadian Health Libraries Association	
Charleston Conference directors & Against the Grain blog	
	Colleague/friend
DC+MED Google group	
Digital Commons Google group	Digital Commons group
ERIL-L	
HSLI- Health Science Librarians of Illinois	
IR Managers Forum	IR Managers group
LAMPHHS - Librarians and Archivists in the History of the Health Sciences	
LibLicense	
MDMLG - Metro Detroit Medical Library Group	
MedLib-L	
Midwest MLA	
MLA caucus/group	

NAHSL	
NASIG	
NNLM region	
Other	Other
Samvera IR Admin Google Group	
SLA	
Social media	
WHSLA - Wisconsin Health Science Libraries	

Q4 - How satisfied were you with the registration process for MIRL Symposium 2021/2022?

2021	2022
Extremely satisfied	Extremely satisfied
Moderately satisfied	Moderately satisfied
Slightly satisfied	Slightly satisfied
Neither satisfied nor dissatisfied	Neither satisfied nor dissatisfied
Slightly dissatisfied	Slightly dissatisfied

Q5 - Do you have any additional feedback on how registration could/can be improved? [open response]

Q6 - Do you have any comments/feedback on the presentations? Was the content what you expected from an IR symposium? [open response]

Q7 - Were there sessions you particularly enjoyed? Were there sessions you would have left out? [open response]

Q8a - Do you feel enough time was allotted to the amount of content provided?

2021	2022
Far too much	Far too much
Moderately too much	Moderately too much

Slightly too much	Slightly too much
Neither too much nor too little	Neither too much nor too little
Slightly too little	Slightly too little
Moderately too little	Moderately too little
Far too little	Far too little

Q8b- What knowledge did you gain from today? What action can you take today after attending this conference? [open response] (2022 only)

Q9 - Do you have additional feedback about the symposium schedule? [open response]

Q10 - Please let us know if you have any comments or suggestions for future session topics [open response]

Q11 - How did you find Zoom (2021)/WebEx (2022) as a platform for MIRL Symposium?

2021 (Zoom)	2022 (WebEx)
Extremely good	Extremely good
Somewhat good	Somewhat good
Neither good nor bad	Neither good nor bad
Somewhat bad	Somewhat bad
Extremely bad	Extremely bad

Q12 - Do you have any specific comments about the platform? [open response]

Q13 - Did you find the resources on the MIRL webpage useful?

2021	2022
Extremely good	Extremely good
Somewhat good	Somewhat good
Neither good nor bad	Neither good nor bad

Somewhat bad	Somewhat bad
Extremely bad	Extremely bad
I did not use the MIRL website	I did not use the MIRL website

Q14 - What is your opinion about future MIRL meetings? Check all that apply.

2021	2022
Hold annually	Hold annually
Hold biannually	Hold biannually
Please keep future meetings virtual only	Please keep future meetings virtual only
Please consider in-person (when safe to do so) with virtual simulcast	Please consider in-person with virtual simulcast

Q15 – Please let us know if you are interested in volunteering for a future MIRL event (please provide name and email) [open response]

Q16 - Do you have any final thoughts about MIRL 2021/2022? [open response]
